# Blood glutamate scavengers prolong the survival of rats and mice with brain-implanted gliomas

**DOI:** 10.1007/s10637-012-9794-x

**Published:** 2012-02-02

**Authors:** Angela Ruban, Tamara Berkutzki, Itzik Cooper, Boaz Mohar, Vivian I. Teichberg

**Affiliations:** 1 Department of Neurobiology, The Weizmann Institute of Science, Rehovot, 76100 Israel; 2 Department of Veterinary Resources, The Weizmann Institute of Science, Rehovot, 76100 Israel

**Keywords:** Blood glutamate scavengers, Gliomas, Glutamate-oxaloacetate transferase

## Abstract

L-Glutamate (Glu) plays a crucial role in the growth of malignant gliomas. We have established the feasibility of accelerating a naturally occurring brain to-blood Glu efflux by decreasing blood Glu levels with intravenous oxaloacetate, the respective Glu co-substrate of the blood resident enzyme humane glutamate–oxaloacetate transaminase (hGOT). We wished to demonstrate that blood Glu scavenging provides neuroprotection in the case of glioma. We now describe the neuroprotective effects of blood Glu scavenging in a fatal condition such as brain-implanted C6 glioma in rats and brain-implanted human U87 MG glioma in nude mice. Rat (C-6) or human (U87) glioma cells were grafted stereotactically in the brain of rats or mice. After development of tumors, the animals were drinking oxaloacetate with or without injections of hGOT. In addition, mice were treated with combination treatment, which included drinking oxaloacetate with intracutaneous injections of hGOT and intraperitoneal injection of Temozolomide. Animals drinking oxaloacetate with or without injections of hGOT displayed a smaller tumor volume, reduced invasiveness and prolonged survival than control animals drinking saline. These effects were significantly enhanced by Temozolomide in mice, which increased survival by 237%. This is the first demonstration of blood Glu scavenging in brain cancer, and because of its safety, is likely to be of clinical significance for the future treatment of human gliomas. As we demonstrated, the blood glutamate scavenging treatment in combination with TMZ could be a good candidate or as an alternative treatment to the patients that do not respond to TMZ.

## Introduction

In the last few years, an ever increasing body of data have suggested that L-Glutamate (Glu) plays a crucial role in the growth of malignant gliomas, their invasiveness and ability to destroy neighboring brain tissue while being also the possible cause of the tumor-associated seizures that often occur in conjunction with gliomas [1–4].

Studies with glioma cells in culture have shown that the cells release massive amounts of Glu resulting in elevations of the extracellular concentrations of Glu in excess of 100 μM within hours in a space that is 1,000-fold larger than the cellular volume [5]. Significant increases were also demonstrated in the peritumoral space surrounding experimental brain tumors in rats [6] and in malignant gliomas and oligodendrogliomas in human patients [7, 8]. Glu release occurs via system X(c), a Glu-cystine exchanger that releases Glu in exchange for cystine [9], which is used for the synthesis of the cellular antioxidant glutathione. The latter protects tumor cells from endogenously produced reactive oxygen and nitrogen species and also endows tumors with an enhanced resistance to radiation and chemotherapy.

In addition to the role of Glu-cystine exchanger, antagonists of the NMDA and AMPA receptors, two major subtypes of Glu receptors, have been found to inhibit the proliferation and migration of both rat [10] and human [11] malignant gliomas while an antagonist of the Glu metabotropic receptor inhibits Glu release from glioma cells and prevents neurotoxicity [12].

Since gliomas appear to co-opt Glu, its exchangers and receptors [11] in support of their invasive migration, they can be added to the long list of neurological diseases in which Glu excitotoxicity is the common destructive pathway. Accordingly, the therapeutic strategies that have been proposed to counteract the Glu-supporting effects on glioma invasiveness which are based on the use of Glu release inhibitors and Glu receptor antagonists [11, 13, 14] are precisely the same as those proposed for the treatment of acute neurodegenerative disorders involving excess Glu in brain such as stroke or head trauma.

This might appear to be a very promising anti-tumor therapeutic avenue to follow but for the fact that, though very effective in preventing or limiting the neuronal degenerative process in animal models of ischemia and head trauma, the Glu receptors antagonists were found to produce unacceptable side effects or even lethal effects in human clinical trials [15].

The alternative therapeutic strategy based on blood Glu scavenging that is proposed here offers a new and safe avenue for coping with the excess peritumoral Glu and its deleterious effects.

### Blood Glu scavenging as a neuroprotective therapy

The maintenance of brain extracellular Glu at levels below its excitotoxic threshold is performed not only by the Glu transporters located on glia and neurons but also by those present on the antiluminal side of the brain capillary endothelial cells [16, 17]. These transporters remove the excess extracellular brain Glu into blood [18, 19].

We have established [19] the feasibility of accelerating this naturally occurring brain to-blood Glu efflux by decreasing blood Glu levels with intravenous oxaloacetate (OxAc) or pyruvate, the respective Glu co-substrates of the blood resident enzymes glutamate–oxaloacetate transaminase (GOT) and glutamate–pyruvate transaminase (GPT). We later demonstrated that blood Glu scavenging provides highly significant brain neuroprotection since both OxAc and pyruvate improve the neurological status of rats submitted to close head injury [20–22].

The latter papers lacked the direct evidence that blood Glu scavenging is the direct cause of a decrease of Glu in the brain fluids in areas where it is in excess. The evidence for that, was recently provided for the first time by a very recent paper of Campos et al., 2011 [23]. In an animal model of middle cerebral arterial occlusion (MCAO) following the Stroke Therapy Academic Industry Roundtable (STAIR) group guidelines [24], oxaloacetate-mediated GOT activation was found to inhibit the increase of blood and cerebral Glu, to reduce the infarct size, the edema volume, and lower sensorimotor deficits with respect to controls. Magnetic resonance spectroscopy established that the excess of Glu levels in the infracted brain parenchyma decreased in parallel with the decrease of blood Glu levels [23].

In two clinical independent studies including more than 350 stroke patients, Campos et al. 2011 [25] found that patients with poor outcome and larger infarct volume at three months of hospitalization show higher Glu [26] and lower GOT levels in blood at the time of hospital admission as compared to those patients with good outcome that had higher blood GOT levels. These findings of Campos et al. (2011) not only bring the final missing proofs for the neuroprotective mechanism of blood Glu scavenging, but also suggest that a treatment to increase GOT levels and decrease Glu in blood, will show effectiveness for neuroprotection in brain pathologies where Glu is in excess.

### Blood Glu scavenging as a chronic therapy

So far, the optimal blood Glu scavenging treatment for the acute neurodegenerative conditions has consisted of a 30 min long intravenous administration of OxAc and recombinant GOT. This combination is required both for the administration of realistic concentrations of OxAc and for the fact that the basal GOT levels in blood are limiting [22]. In our previous studies, we intravenously injected rats with OxAc to reach a steady state concentration in blood [19]. However, one can maintain (as expected from the Michaelis-Menten equation) the same rate of blood Glu transamination by reducing significantly the concentration of the administered OxAc while increasing in parallel the GOT blood concentration. Using this approach, we found [22] that an optimal blood Glu scavenging and neuroprotection after TBI could be attained by administering 1 ml of 10 mM OxAc/100 g rat (this dose is ineffective on its own) together with 13 μg recombinant GOT. Increasing the GOT concentration in blood facilitates the OxAc-mediated blood Glu scavenging since it allows the intravenously administered OxAc to readily react with the transaminase before being transported into the various body organs via the dicarboxylate transporters [27, 28].

For studying a chronic disease such as glioma, we relied on the endogenous blood levels of GOT for OxAc-mediated blood Glu scavenging and the treatment modality we used was based on restricting the rats to a drink of 0.2 M OxAc in tap water. The above oral treatment probably should be modified for the clinical trials since OxAc is subjected to a first-pass metabolism in the liver as well as an absorption into the gut wall on its possible way to the portal vein. Accordingly, the plausible mechanism for blood Glu scavenging could be based on a GOT-mediated transamination reaction taking place not only in blood but also in the liver leading to a reduced Glu output from the liver into blood [29].

In the present study we investigated whether blood Glu scavenging could be used in experimental glioma to reduce the brain levels of excess Glu that are continuously released by the tumor cells. Our results demonstrate the therapeutic effects of blood Glu scavenging in a C6 glioma rat model as well as in the human U87 MG glioma model in nude mice.

## Materials and methods

### Materials

Rat C6 glioma and human U-87 Glioma cells were obtained from ATCC. The tumor cell lines were routinely tested for authenticity by microsatellite analysis, at the ATCC. Oxaloacetic acid was obtained from Sigma. His-tagged human Glutamate-Oxaloacetate transaminase (hGOT) cDNA was cloned from the human hepatoma cell line hepG2, expressed in *E. coli* and purified by Ni-agarose chromatography. Temozolomide was obtained from Schering Corporation. A stock solution of temozolomide (12.5 mg/ml) was prepared in 100% DMSO, and then filtered through a 0.22 μm filter. 1 ml aliquots were placed in sterile Eppendorf tubes and stored at −70°C. Immediately prior to injection, the stock drug solution was thawed, diluted in sterile saline to a final concentration of 0.125 mg/ml. 5 mg/kg Temozolomide was injected intraperitoneally at day 7 post tumor implantation in a volume of 1 ml saline/ 1% DMSO (Temozolomide dose: 15 mg/m^2^ and 120 ml/m^2^).

### Animals

The experiments were conducted according to the recommendations of the declarations of Helsinki and Tokyo and to the Guidelines for the Use of Experimental Animals of the European Community approved by the Animal Care Committees of Weizmann Institute of Science. The experiments with C6 glioma were carried out on male rats of the Sprague–Dawley strain, weighing 250–300 g (Harlan Biotech, Israel). The experiments with U87 MG were carried out in male CD1 nude mice, 9–10 weeks (Harlan Biotech, Israel). Purina Chow and water were available ad libitum. Ambient temperature was set to 22–23°C.

### Intracerebral injections of C6-glioma

Rats were anesthetized with 1.5% Halothane (Rhodia) solution by a respiration system and immobilized on a stereotaxic unit. After disinfection and incision of the skin, C6 cells were stereotaxically implanted in the left anterior corpus striatum using the following coordinates: 0 mm bregma, +3 mm left lateral, and 5.5 mm depth. C6 cells were resuspended in PBS (10^4^ cells/2 μl) and implanted at an infusion rate of 1 μl/10 min. The needle was left in place 5 min after cell infusion and it was slowly withdrawn. All tumor cell implantations were performed using a Hamilton syringe with a 26-gauge needle (Hamilton Co.) attached to the stereotaxic system.

Seven days after the tumor cell implantation, rats were randomly divided into two therapeutic groups (*n* = 14 for control group and *n* = 15 for treated group). The treated group was provided daily with a freshly-prepared 0.2 M OxAc solution in water. The pH was adjusted to 7.0–7.4 with 1 M NaOH. Since the OxAc solution included Na^+^ molecules originals from the neutralizing NaOH, the control group was provided with NaCl (0.3 M) solution as their drinking water. Rats were monitored daily to detect any signs of neurological suffering from tumor growth or from toxicity effects of the therapy. All the rats survived until the end of the experiment and were sacrificed at day 21–22 post tumor implantation (PID).

### Magnetic resonance imaging

High resolution brain MRI was performed twice using T2 and T1-weighted imaging before and after injection of 0.4 ml contrast medium (Gadodiamide, Omniscan, Amersham) into the tail vein. The animals were examined in a 4.7 Tesla (200 MHz, 1 H) magnet with a 40 cm inner diameter bore system (Bruker Biospec, Ettlingen, Germany). A birdcage radiofrequency (RF) coil with an inner diameter of 70 mm was used. In vivo multi-slice images of rat brains were acquired in the coronal plane (ten slices; slice thickness 1.0 mm; matrix = 128 × 128, field of view = 32 × 32mm2).T2W images were acquired using a fast-spin-echo sequence (echo train length = 4; repetition time = 3 s; echo time = 64 ms; number of averages = 2). T1w images (repetition time = 700 ms; echo time = 10 ms; number of average = 10) with and without gadolinium enhancement were acquired with the same geometry and location as the T2w image. Gadodiamide-enhanced T1 imaging was performed 5 min after injection. During MRI examination, rats were anesthetized by isoflurane (initial dose 2.5%, maintenance 1.6%). To assess the tumor size, rats were submitted to MRI at day 7 PID. After 14 days of treatment (21 PID), rats were submitted again to MRI to establish the extent of tumor growth estimated using serial T1-W and T2-W spin-echo sequence images evaluated with the Surf driver program. Tumor volumes were determined using MRIcro software At 7 PID T1 and T2 imaging were performed as a volume of the tumor was small and the borders were poorly detectable in the T2 imaging. At 21 PID only T2 imaging was performed as a volume of the tumor was big and the shorter anesthesia time kept all the animals alive up to the end of the procedure.

### U87 MG cell intracerebral implantation and treatment

Orthotopic glioblastoma-bearing mice were obtained by an intracranial implantation of 25 × 10^5^ U87 MG cells in 10-weeks-old male immunodeficient CD1 nude mice under xylazine (10 mg/kg)/ketamine (70 mg/kg), anesthesia. After disinfection and incision of the skin, U87 cells were stereotaxically implanted using the following coordinates: 0.5 mm forward from the bregma, 2.1 mm lateral, and 3.0 mm ventral from the dura. Seven days after tumor cell implantation, mice were randomly divided into five groups (*n* = 13/group, totally 65 mice).

Group 1 was a control group of CD1 nude mice to which, starting on day 7 post tumor implantation, a subcutaneous administration of 200 μl saline was performed every other day until death. Starting from day 7 and on four consecutive days, mice were injected intraperitoneally with 1 ml PBS/1% DMSO and until the termination of the experiment, mice were supplied with drinking water containing of 0.3 M NaCl in tap water.

Group 2 was a treatment group of mice to which, starting on day 7 post tumor implantation, a priming dose of hGOT 2.14 mg/kg was administered subcutaneously in 200 μl saline followed by maintenance doses of hGOT 0.214 mg/kg in 200 μl saline performed every other day until death. On day 7 and on four consecutive days, mice were injected intraperitoneally with 1 ml saline/1% DMSO. Starting on day 7, until the termination of the experiment, mice were offered drinking water consisting of 0.2 M OxAc in tap water.

Group 3 was a treatment group of mice to which TMZ was administered. Starting on day 7 post tumor implantation, subcutaneous administrations of 200 μl saline were performed every other day until death. Also, starting from day 7 and on four consecutive days, mice were injected intraperitoneally with 1 ml saline/1% DMSO containing 5 mg/kg of TMZ. Until the termination of the experiment, mice were supplied with drinking water consisting of 0.3 M NaCl in tap water.

Group 4 is a combination treatment with TMZ/OxAc/hGOT. Starting on day 7 post tumor implantation, a priming dose of hGOT 2.14 mg/kg was administered subcutaneously in 200 μl saline followed by maintenance doses of 0.214 mg/kg in 200 μl saline performed every other day until the termination of the experiment. On day 7 and on four consecutive days, mice were injected intraperitoneally with 1 ml saline/1% DMSO containing 5 mg/kg of TMZ. Starting on day 7 and until death, mice were offered drinking water consisting of 0.2 M OxAc in tap water.

Mice were monitored daily to detect any signs of neurological suffering from tumor growth or from toxicity effects of the therapy. At day 14, the mice were deeply anesthetized with urethane (1.3 g/kg, i.p.) and rapidly perfused transcardially with 50 ml of 0.1 M phosphate buffer, (pH 7.4) containing 4% paraformaldehyde (Sigma-Aldrich, Israel; BioLab, Israel). Brains were removed and the hemisphere containing the tumor, as well as the contralateral hemisphere (for control), were postfixed in the same fixative overnight. Tumor volume was estimated by histology analysis.

### Histology and immunohistochemistry

At indicated time points, the animals were anesthetized and perfused transcardially with chilled PBS, brains were removed and fixed in Bouin fixative or in 4% formalin. Samples were processed for paraffin embedding by standard procedure. Coronal brain serial sections 6 μm thick were generated with spacing 500 micron between series. All sections which included glioma cells were used for histology and immunohistochemistry. From each sectional level four slides were generated. One of them was stained by hematoxylin and eosin (H&E) and the adjacent three slides were used for immunohistochemistry For immunostaining, after deaxing and hydration of sections, antigen retrieval was done in citrate buffer (pH 6.0) using a microwave technique. Endogenous peroxidase was blocked and the sections were incubated overnight with primary antibody at 4°C. The PC-10 monoclonal antibody to proliferative nuclear antigen ( PCNA) (BioLegend, San Diego,CA) diluted 1:1,000, a cocktail of mouse monoclonal antibody to glial fibrillary acid protein (GFAP) (BD Bioscience) diluted 1:200 and goat anti Aurora Kinase A/AURKA antibody diluted 1:50 (Sigma) were used. The antibody binding was visualized using streptavidin-peroxidase complex Histofine (Nichirei, Tokyo, Japan). Peroxidase activity was revealed with 0.01% hydrogen peroxide, using 3,3′-diaminobenzidine as the chromogen (Sigma). Known positive controls and negative controls were included in each batch.

### Quantitative evaluation

Sections were observed and captured through an optical Eclipse E800 microscope (Nikon, Japan) and a digital camera DMX 1200 (Nikon,Japan). ImagePro Plus software (Media Cybernetics, Silver Spring, MD) was used for quantitative analysis. Volume of tumor was estimated by three dimensional reconstructions after tracing of tumor contour on the images of H&E stained serial sections. The number of tumor cell nuclei per 10,000 micron was calculated in 10 microscopic fields of each sample (objective magnification ×40), allowing treatment effects to be tested on tumor cellularity. Proliferative activity in tumor tissue was evaluated on the slides stained for PCNA by counting immune-positive nuclei and immune-negative tumor cell nuclei in 10 microscopic fields of each sample (objective magnification ×40) and expressed as percent of immune-positive nuclei to the total nuclei counted. Astrocytes were identified by using GFAP immunohistochemistry. The quantification of GFAP-positive cells was performed on the images captured from the field of brain tissue around the tumor by objective magnification ×40 for assessment glial scar density around the tumor. At least five images in each case were used for analysis. Number of stained cells per area was used for assessment of glial scar density around the tumor. All morphometric analyses were performed without previous knowledge of the experimental group from which the sections were obtained.

### Survival assay

We repeated the therapy with intracranial U87 MG xenografted mice, comparing the four groups on a survival basis. Orthotopic glioblastoma-bearing mice were obtained by an intracranial implantation of 25 × 10^5^ U87 MG cells in 10-week-old male CD1 nude mice. Seven days after the tumor cell implantation, mice were randomly divided into four therapeutic groups (*n* = 13/group). Schedule and doses of therapeutics were the same as mentioned above. Mice were monitored daily and euthanized at the appearance of severe neurological damage from tumor growth.

### Statistics

Statistics were calculated using Prism version 4.0 (GraphPad software). Student’s *t*-Test (two-tailed) was used to look for significant differences between cell number in C6 cells exposed to OxAc, MRI analysis of intrastriatal infusion of C6 cells and viability increase in U87 cells exposed to OxAc. One-way ANOVA with Newman-Keuls post test was used to compare tumor growth in CD1 nude mice. Kaplan-Meier survival graphs were analyzed by Log-rank test. In all tests, statistical significance was assumed for *p* < 0.05.

## Results

### The effect on tumor development following the chronic administration of 0.2 M OxAc in the drinking solution of C6-glioma implanted rats

**Fig. 1:**
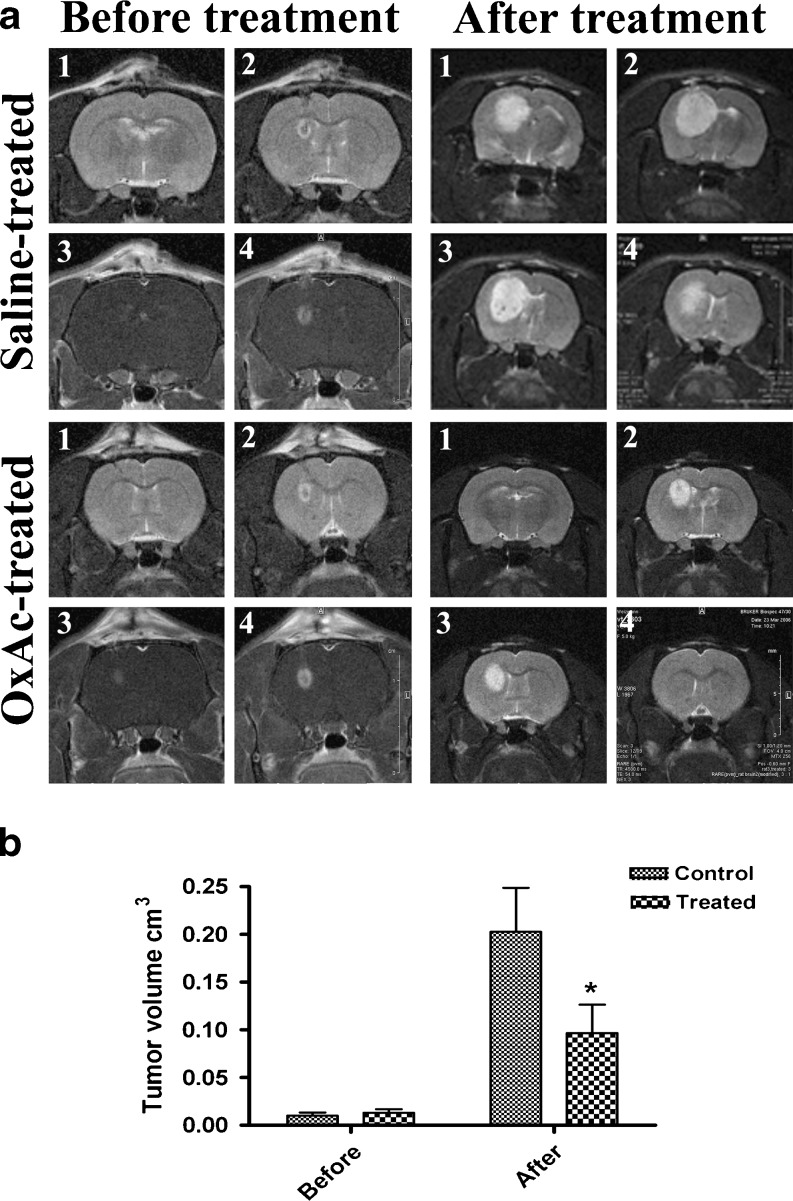
**a**. A representative example of MRI analysis of C6 glioma growth in vivo. At 7 days post-implantation and before the treatment, an MRI imaging was performed (left column, 1–2 pictures T2 imaging, 3–4 pictures T1 imaging). The rats were randomized and either treated with 0.2 M OxAc or 0.3 M NaCl for additional 14 days. The MRI imaging was performed again at the end of the treatment (21 days post glioma implantation, right column, 1–4 pictures T2 imaging). **b**. Tumor volume of the rats before and after the treatment was calculated in *n* = 14 for control group and in *n* = 15 for the treated group using MRIcro software. OxAc-treated rats show significantly reduced tumor growth compared to the control group. *p < 0.01 (repeated measures ANOVA test)

In order to evaluate the potency of the oral treatment of brain C6 glioma with a blood Glu scavenger such gas OxAc, cultured C6 cells were implanted into the left striatum of rats to induce tumors and the accompanying neovascularization. The tumor development was followed by MRI. Seven days after the cell implantation, as the presence of a growing tumor mass was confirmed by MRI (Fig. 1a. left column), the rats (*n* = 15) were offered a drink with a 0.2 M OxAc solution as the oral solution while the control rats (*n* = 14) were offered a liquid containing of 0.3 M NaCl. MRI analysis shows that OxAc-treated rats significantly reduced tumor growth as compared to the control group. (*p* < 0.01, repeated measures ANOVA, Fig. 1a. right column and b).

### Blood glutamate scavenging in combination with TMZ inhibits tumor growth in an orthotopic CD1 nude glioma model

**Fig. 2:**
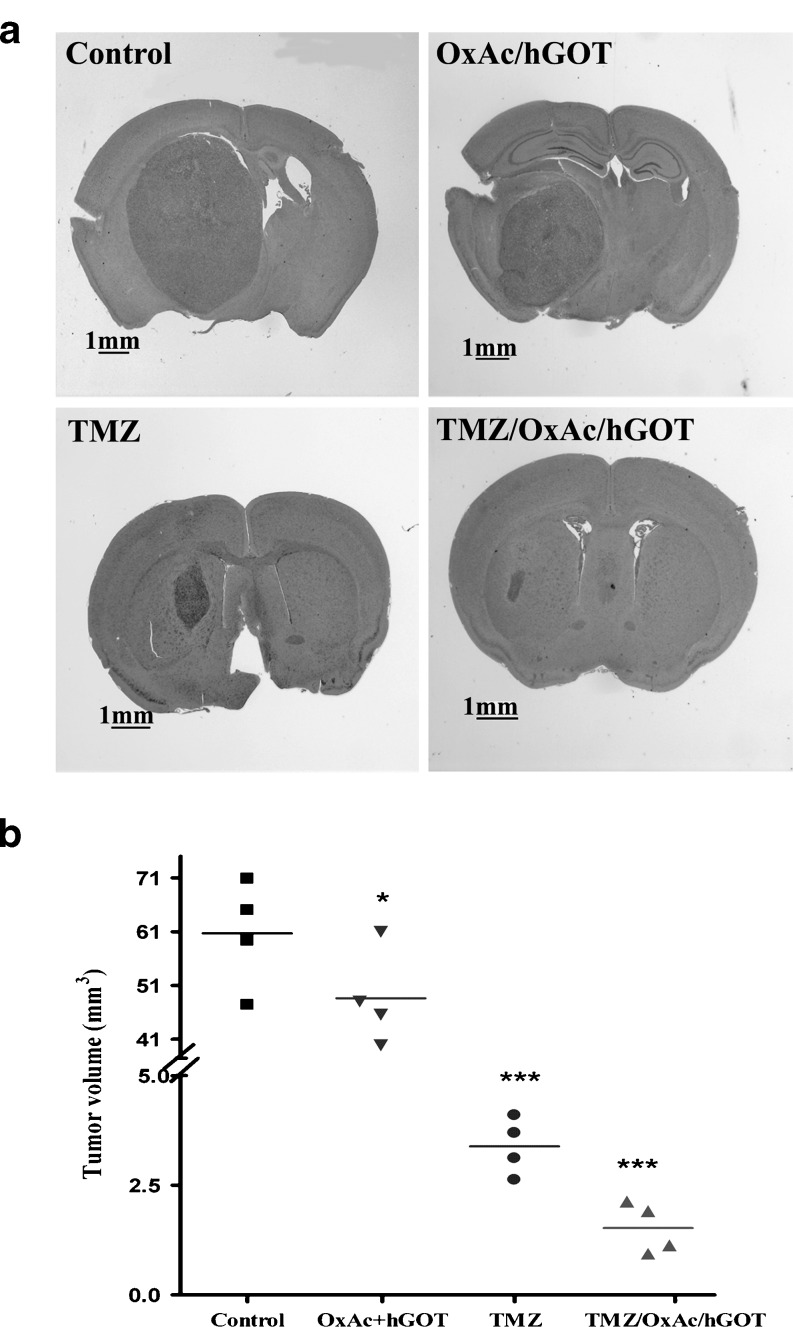
Tumor growth over 21 days in CD1 nude mice. U87 GM bearing CD1 nude mice were treated on day 7 with OxAc+hGOT, TMZ, OxAc+ hGOT +TMZ combined treatment, and control. *p < 0.05; ***p < 0.001 (one-way ANOVA with Newman-Keuls post test, *n* = 4) versus control group treated with 0.3 M NaCl

On the strength of the fact that the administration of exogenous GOT transforms an ineffective low dose of OxAc into an effective one [22], we added hGOT to the treatment in order to elevate the blood GOT levels and possibly improve the effectiveness of oral OxAc for the scavenging of blood Glu. As temozolomide (TMZ) is a standard treatment of glioma, we also determined the anti-tumor effects of OxAc/hGOT, TMZ and their combination. Tumor size was monitored in the orthotopic nude mice glioma model after 14 days of treatment i.e. 1 week post tumor implantation. The mean tumor volumes ± SD were 61 ± 10 mm^3^, 47 ± 9 mm^3^ , 3.5 ± 0.5 and 1.5 ± 0.6 mm^3^ for the NaCl-treated, OxAc/hGOT, TMZ and TMZ/OxAc/hGOT groups respectively (Fig. 2).

### Histology of the U87 MG glioma in nude mice

**Fig. 3:**
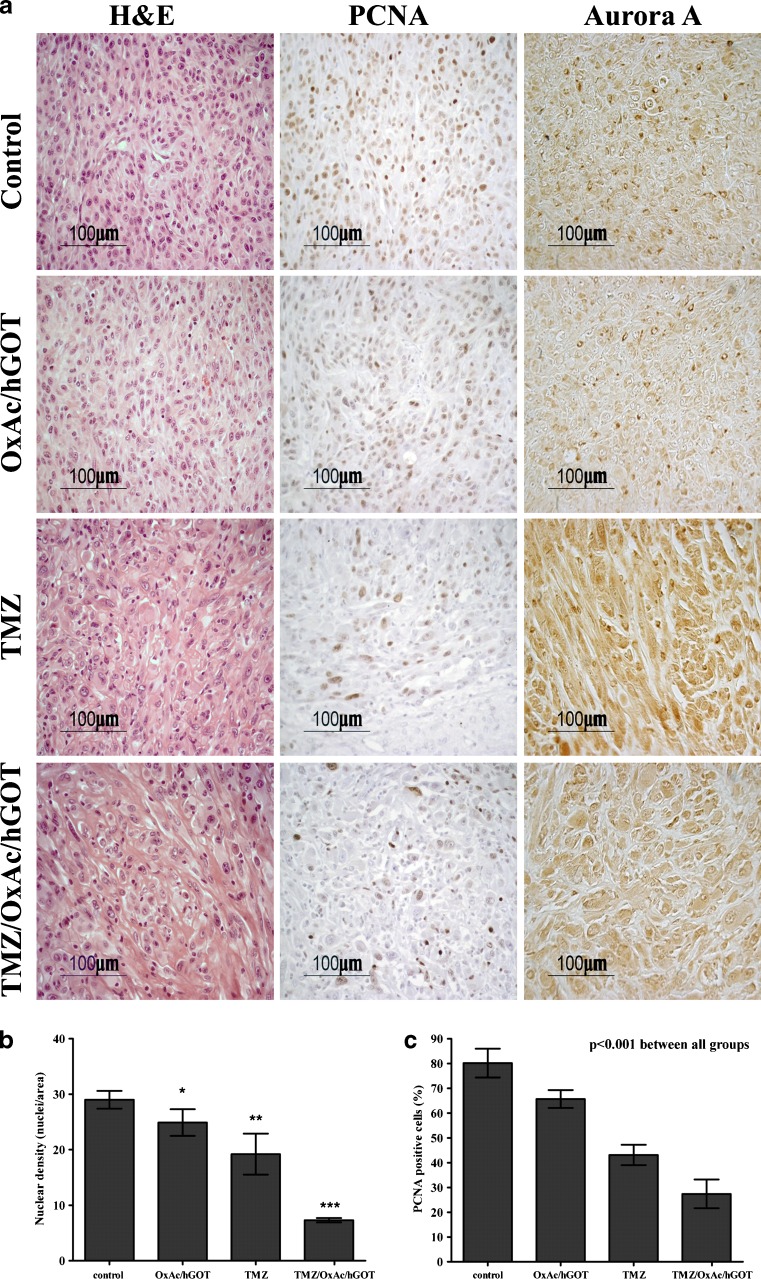
Morphology of tumors derived from U87 glioma cell line A. Immunohistochemistry for H&E, PCNA and Aurora A. Data are representative of four mice per group. B. Quantification of tumor cell nuclear density (H&E staining); *p < 0.05, **p < 0.001 vs. control group, ***p < 0.001 vs. all other groups (one-way ANOVA+ Newman-Keuls test, *n* = 4. C. Quantification of the PCNA positive tumor cell nuclei staining; one-way ANOVA revealed significant differences between all groups (*p* < 0.001). Data presented as mean values ± SD

To characterize the U87 MG tumor in vivo, in addition to the tumor volume monitoring, we performed standard immunohistological staining focusing first on the tumor center:

1) H&E staining shows that in NaCl-treated mice tumors show high nuclear pleomorphism and nuclear density (mean nuclear density: 29 ± 1.6). These characteristics are significantly less pronounced in the OxAc/hGOT-treated group (24.9 ± 2.4), in the TMZ group (19.2 ± 3.7) and in the TMZ/OxAc/hGOT combination group (7.3 ± 0.4; *p* < 0.001 with respect to the TMZ-treated group. (Fig. 3a left panel and b).

2) The PCNA data show a significant reduction in DNA synthesis i.e. in tumor cells proliferation, in all treated groups versus control (% PCNA positive cells: NaCl: 80.2 ± 5.8; OxAc/hGOT: 65.7 ± 3.6; TMZ: 43.1 ± 4.1 and TMZ/OxAc/hGOT: 27.4 ± 5.8; (Fig. 3a middle panel and c). Thus, the most significantly proliferative reduction was shown in the TMZ/OxAc/hGOT combination treated group.

3) We used the Aurora A staining as a marker for grading the glioma tumor. Aurora A, which is also known as serine/threonine kinase 15 (STK15), is a key regulatory protein controlling centrosome maturation, spindle assembly, and chromosome segregation [30]. The presence of high Aurora A expression levels were shown to correspond with nuclear and centrosomal abnormalities in high-grade gliomas [31]. In the current study, reliable quantification of the Aurora A staining was very hard to obtain due to very significant morphological differences between the groups, which reflected in high background for the staining. However, it is quite obvious that there is a trend to lower tumor grade in the treated groups with almost no positive staining observed in the TMZ/OxAc/hGOT combination group (Fig. 3a right panel, small dark brown spots indicate positive staining).

**Fig. 4:**
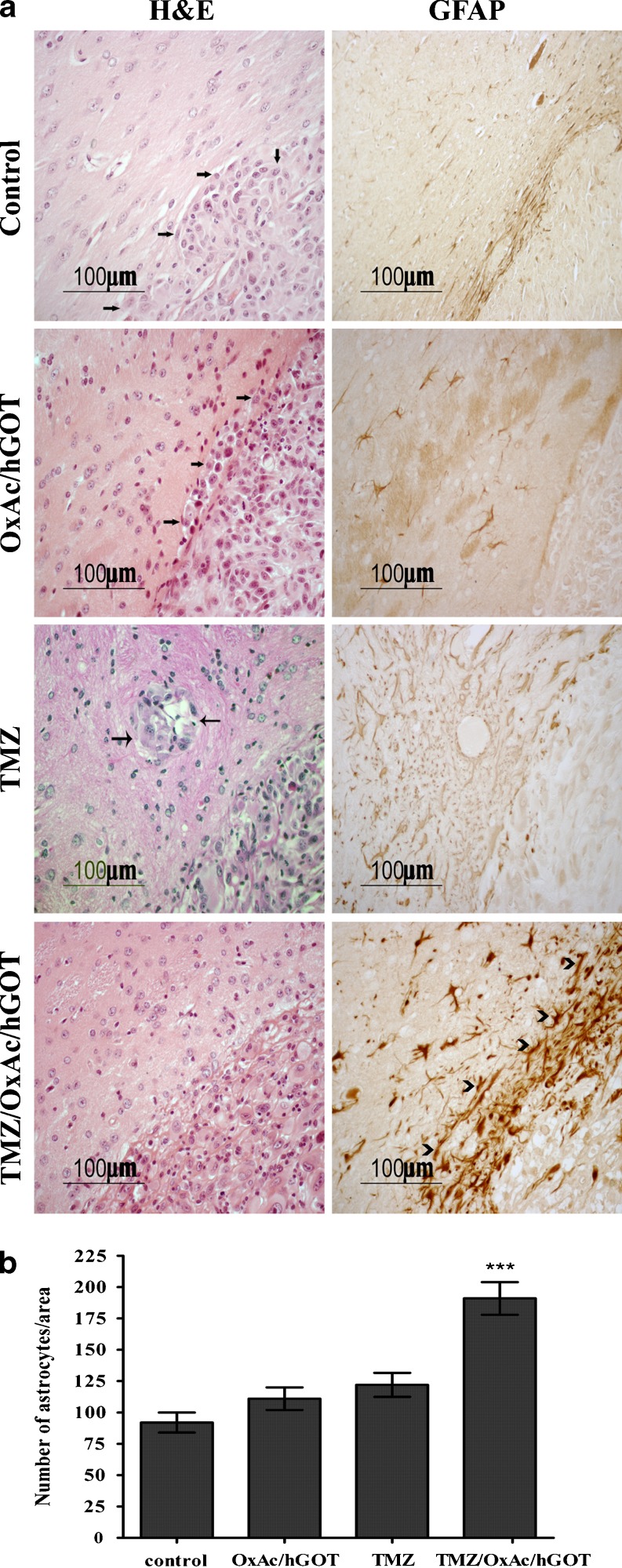
Morphological evidence of the tumor invasiveness derived from U87 glioma cell line. **a**. Representative example of immunohistochemistry staining for H&E and GFAP. Staining by H&E showing infiltration of the brain parenchyma by tumor cells detached from the main tumor mass, arrows indicate the infiltrative cells (left column). Staining for GFAP showing the astrocytes cells around the tumor borders, arrowheads indicate the GFAP positive astrocytes (right column) **b**. Quantification of the number of astrocytes as indicated by positive GFAP staining,; ***p < 0.001 vs. all other groups, One-way ANOVA+ Newman-Keuls test, *n* = 4

Analysis of the tumor invasiveness using H&E immunostaining shows a significant tumor cell infiltration into the brain parenchyma around the borders of the tumor in the control group and in the OxAc/hGOT and TMZ-treated groups (see arrows in Fig. 4 left panel). This was however absent in the TMZ/OxAc/hGOT-treated group probably as the result of the conspicuous astrocytic scar revealed by GFAP immunostaining (Fig. 4, right panel, see arrowheads). One can assume that the astrocytes surrounding the borders of the tumor prevent the tumor growth and invasiveness (number of astrocytes/area: 191 ± 33; *p* < 0.001, One-way ANOVA+ Newman-Keuls test). This was not observed in all other treated animals (124 ± 3, 110 ± 3 and 93 ± 2 for TMZ, OxAc/hGOT and saline groups, respectively).

### Increased survival of CD1 nude mice treated with blood glutamate scavengers with or without TMZ

**Fig. 5:**
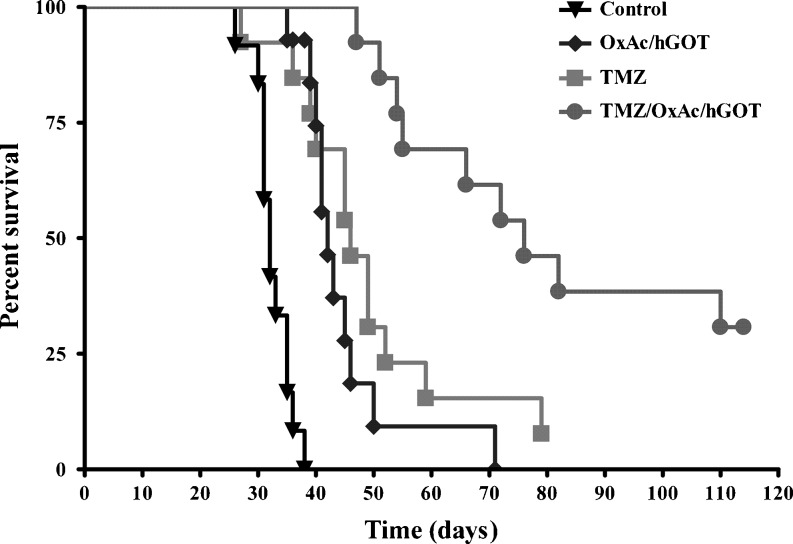
Survival rate in CD1 nude mice-bearing intracranial U87 MG cells. Kaplan-Meier survival of CD1 nude mice treated with 0.3 M NaCl 50 ml/day (*n* = 13), OxAc 2.14 mg/kg/day/hGOT 2.14 mg/kg on the first day followed by a maintenance dose of 0. 214 mg hGOT/kg/day (*n* = 13), TMZ 5 mg/kg for 4 days (*n* = 13) and a combined TMZ/ OxAc/hGOT treatment with the same doses as the rest groups (*n* = 13). Animals were monitored up to 115 days after the tumor cell implantation. P-Values obtained using a Long-rank test show *P* < 0.0001

A survival study was conducted in separate groups of mice (Fig. 5). The median survival time in the vehicle (DMSO/saline)-treated animals was 32 days (*n* = 13), 42 days in the OxAc/hGOT-treated animals (*n* = 13), 46 days in the TMZ-treated animals (*n* = 13) and 76 days in the TMZ/OxAc/hGOT-treated animals (*n* = 13). One of the TMZ-treated mouse and four of the combination treatment mice continue to survive >115 days after tumor implantation. All the experimental animals were treated until death or until they were sacrificed. These mice were sacrificed at day 117 and their brains were analyzed for the presence of tumor cells. No tumor cells were found (data not shown). The survival of mice in the treated groups vs. control was prolonged by 137%, 144% and 237% for the OxAc/hGOT, TMZ and TMZ/OxAc/hGOT combination treatment groups respectively (*p* < 0.0001), exhibiting again the advantage of the combination treatment on the other groups.

## Discussion

Treatment of primary central nervous system tumors such as gliomas includes surgery, radiation and chemotherapy with agents such as TMZ, BCNU or 1-(2-chloroethyl)-3-cyclohexyl-L-nitrosourea combined with vincristine [32]. Unfortunately, even the combined modality therapy yields only 2% to 5% five-year survival rates, and poor treatment response. The dismal prognosis is due to postsurgery recurrences arising from escaped invasive tumor cells.

This manuscript describes for the first time the ability of blood Glu scavengers to enhance the treatment at gliomas. This ability stems from the fact that excess Glu in the peritumoral space of gliomas plays a crucial role in glioma invasiveness. Glu released via the Glu-Cystine exchanger kills neighboring neurons and allows the tumor to occupy an ever increasing space within the brain [2–4]. Our results demonstrate that treatment with blood Glu scavengers clearly contributes to the inhibition of the glioma invasiveness as well as to the survival of the treated animals.

In the rat model of C6 glioma, animals treated by OxAc show a significantly reduced tumor growth compared to the control group (Fig. 1). To exclude the possibility of a direct toxic effect of the OxAc treatment on glioma cells, a 3-(4,5-dimethyltiazol-2yl)-2,5-phenyl-2H-tetrazolium bromide (MTT) assay was performed on C6 cells exposed in-vitro to OxAc (3 μM–0.3 μM) for 24 h and no effect was found (data not shown). Considering our previous [4, 19–21] and current results using blood Glutamate scavenging in several animal models we suggest that the therapeutic effects of OxAc on glioma growth and invasiveness in vivo are due primarily to the scavenging effect of OxAc blood Glu. In the case of glioma, our results strengthen the concept that OxAc caused in increased efflux of peritumor Glu into the blood, slowing down the ability of the tumor to create the space for its expansion.

Having demonstrated the in vivo effectiveness of OxAc to decrease rat glioma growth, we investigated the effects of OxAc/hGOT alone and/or with TMZ treatment on human glioma cell line implanted in nude mice. The hGOT was added to the treatment in order to increase the blood GOT levels and possibly improve the efficiency of the oral OxAc for the scavenging of blood Glu. We showed that the combination treatment had an improved effect on tumor growth inhibition compared to TMZ alone (Fig. 2). The OxAc/hGOT treatment had a statistically significant effect on tumor growth, but less impressive than TMZ alone or the combination treatment. In spite of the statistically insignificant difference in tumor volume between TMZ and OxAc/hGOT/TMZ groups there is a clear trend of improvement after the combination treatment. Using a larger number of animals, would most likely have made it statistically significant. However, the peripheral administration of OxAc/hGOT contributed in a very significant way to the survival of TMZ-treated mice (Fig. 5). The most impressive effect on survival duration was demonstrated b they combination treatment.

In a similar way with what was observed with C6 cells, the U87 cells viability was assessed in-vitro after 24 h exposure to OxAc (3 μM–0.3 μM) and no effect was found (data not shown).All immunostaining methods we used confirm the fact that the U87 MG cells in the NaCl-treated Nude mice show both proliferation and invasiveness. Staining with H&E showed infiltration of the brain parenchyma by cells detached from the main tumor mass. This trend is systematically reduced in all mice treated with a blood Glu scavenger (Fig. 4). The most interesting results were obtained by GFAP staining that showed the remarkable recruitment of astrocytes around the borders of the tumor in the combination treated group only. This is the first time that this phenomenon was observed. As already discussed above, excessive amounts of Glu can cause excitotoxicity and hence Glu release from gliomas may similarly exert peritumoral excitotoxicity. Astrocytes are typically resistant to even millimolar concentrations of Glu [33]. As it pertains to peritumoral astrocytes, their response to excess Glu is not well understood. Astrocytes typically respond to neuronally released Glu by the rapid uptake into the cytoplasm using the EAAT1 and EAAT2 transporters. It is clear, however, that the Glu release from the tumor must overwhelm the astrocytic capacity to sequester Glu and maintain proper Glu homeostasis. The space occupied by expanding tumors is devoid of neurons and astrocytes alike. Low-grade glioma contain a lower density of tumor cells intermixed with normal brain, and as these tumors progress to higher grade, thus astrocytes must be killed by the expanding tumor [1]. Indeed, we show that no GFAP positive astrocytes are observed in the control group, as well as in OxAc/GOT or TMZ treated groups (Fig. 4). This strongly supports our histological observations showing a clear trend of blood Glu scavengers to reduce the glioma grade (Fig. 3). It is important to emphasize that despite the significant reduction of tumor volume and extension survival of mice treated by TMZ alone, addition of the glutamate scavengers resulted in a significant improvement in all parameters.

TMZ has received much attention in the last 10 years and become a current standard chemotherapy treatment of malignant gliomas, but patient’s prognosis remains unsatisfactory. The limited efficacy of the chemotherapy can be attributed to both inherent and acquired tumor drug resistance [34, 35]. Thus, there is a need for improving the current therapeutic approach.

## Conclusions

We have shown that the in vivo periodic administration of blood Glu scavengers is highly effective in reducing the proliferation and invasiveness of both rat C6 glioma cells and human U87 MG cells in rat and mice glioma model systems. It is clear that a treatment of such multifactorial disease like glioma ought to be a complex therapy. As we demonstrated in this study, the blood glutamate scavenging treatment could be a good candidate in combination with TMZ or as an alternative treatment to the patients that do not respond to TMZ. This is the first time that this approach is used and it appears that it may be of high clinical significance for the future treatment of gliomas.
